# Trichohepatoenteric syndrome type 1: expanding the clinical spectrum of THES type 1 due to a homozygous variant in the SKIC3 gene

**DOI:** 10.1186/s12887-024-04924-7

**Published:** 2024-07-10

**Authors:** Abdullah Alrammal, Rashed Aljundi, Sedra Abu Ghedda, Buthaina Mustafa AlMurbati

**Affiliations:** 1 University of Sharjah, Sharjah, United Arab Emirates; 2 Faculty of Medicine, University of Aleppo, Aleppo, Syria; 3 Pediatric Department, Al Jalila Children’s Specialty Hospital, Dubai Health, Dubai, United Arab Emirates

**Keywords:** Trichohepatoenteric syndrome, Infantile diarrhea, Mutation, Dysmorphic features, Infantile liver disease

## Abstract

Trichohepatoenteric syndrome (THES), also known as phenotypic diarrhea or syndromic diarrhea, is a rare autosomal recessive genetic disorder caused by mutations in SKIC2 (THES-type 2) or SKIC3 (THES-type 1) and is characterized by early onset diarrhea, woolly brittle hair, facial dysmorphic features and liver disease. We report the case of a 24-month-old girl who presented with chronic diarrhea since the neonatal period along with intrauterine growth restriction (IUGR), developmental delay, dysmorphic features, congenital heart defects, liver disease, and recurrent infections. The diagnosis was made through whole-exome sequencing analysis, which detected a homozygous variant (c.4070del, p.Pro1357Leufs*10) in the SKIC3 gene. The patient required parenteral nutrition and was hospitalized for the first 10 months of life and then discharged on PN after showing improvement. She remained stable on PN after discharge despite a few admissions for central line infections. Recent follow-up at the age of 2 years revealed that she was stable on long-term parenteral nutrition and that she had advanced chronic liver disease.

## Introduction

Trichohepatoenteric syndrome (THES) is a rare autosomal recessive genetic enteropathy with an estimated prevalence of 1/1,000,000 births globally [1]. Studies have shown that there is no relationship between ethnic groups and the incidence of this genetic disorder. A mutation in the SKIC2 gene (formerly known as SKIV2L, which stands for superkillerviralicidic activity 2) is thought to be the cause of THES type 2, while the SKIC3 gene (previously referred to as TTC37, which stands for tetratricopeptide repeat domain-containing protein 37) is believed to be responsible for THES type 1. The 2 genes encode the human superkiller (SKI) complex, which controls the mRNA degradation system. However, it is not fully understood how alterations to the mRNA system result in abnormalities in the clinical picture [2].

This disease presents as a broad spectrum of symptoms characterized mainly by early-onset intractable diarrhea, which leads to failure to thrive and subsequent mental retardation, woolly hair abnormalities, facial dysmorphism, liver disease, intrauterine growth restriction (IUGR), immune dysfunction, congenital heart disease and skin abnormalities [1].

The diagnosis of THES is achieved through the clinical picture, and it is confirmed by genetic testing. Early mortality in untreated patients is mainly related to liver cirrhosis and infections, which is why early detection and management are important for better outcomes [2].

Currently, the management of the majority of SD/THE patients primarily relies on parenteral nutrition and immunoglobulin supplementation. Although antibiotics, steroids, and immunosuppressant drugs have shown limited efficacy, their overall effectiveness remains unsubstantiated [3].

In this report, we present a case of THES type 1 in which whole-exome sequencing (WES) identified a homozygous variant in the SKI3 gene. To our knowledge, the clinical manifestations associated with this variant have not been previously described in the literature.

## Case presentation

A 2-year-old girl presented with chronic diarrhea since the neonatal period. The parents are nonconsanguineous and healthy; she has one older healthy sibling. She was born prematurely at 35 weeks with intrauterine growth restriction (IUGR) and a birth weight of 1430 g. She displayed developmental delay and dysmorphic features, including a wide-open anterior fontanelle, an open metopic suture, a broad forehead, slight facial asymmetry, thin and sparse hair, eyebrows, relatively large ears, thin lips, a downturned angle of the mouth, Café-au-lait spots and bilateral inguinal hernia.

Additionally, congenital cardiac defects, including ventricular septal defect (VSD) and atrial septal defect (ASD), which spontaneously closed 2 months after birth, were noted. Clinical examination also revealed hepatosplenomegaly. She was genetically and clinically diagnosed with THES type 1. Afterward, she was admitted and managed in the neonatal intensive care unit (NICU) as an inpatient for 10 months.

During this period, she exhibited intractable diarrhea resulting in poor growth and electrolyte imbalances. Thus, she required parenteral nutrition (PN). The patient also had decreased levels of immunoglobulins (IgA, IgG, IgM, and IgE) and a reduced absolute lymphocyte count. Moreover, she experienced recurrent urinary tract infections and CMV infections. Prophylactic antibiotics, antifungal agents, and monthly intravenous gadolinium (IVIG) infusions were administered due to her immunodeficiency. She showed improvement and was discharged on PN after 10 months of hospitalization. The patient remained stable on PN after discharge despite a few admissions for central line infections. Additionally, she underwent bilateral inguinal hernia repair without complications.

Since her last admission, the patient exhibited chronic liver disease characterized by portal hypertension and elevated liver enzyme levels, pancytopenia, hypoglycemia with TPN breaks, hypogammaglobulinemia, hypophosphatemia, hypercalcemia, anemia requiring blood transfusions, and proximal renal tubular dysfunction. A liver biopsy revealed portal fibrosis and biliary ductular proliferation. She underwent gastroscopy, which indicated that she had two grade 2 esophageal varices and a fundal varix. A stool test revealed an increase in calprotectin (983 µg/g). Additionally, she had feeding difficulties with oral feeding, which required gastrostomy. The patient received comprehensive care through a multidisciplinary approach. Her management plan included PN for intestinal failure, monitoring and management of the liver, blood transfusions for pancytopenia and anemia, phosphate supplementation for proximal renal tubular dysfunction, propranolol for portal hypertension, nutritional counseling, and developmental therapies. She showed a stable state afterward.

A recent follow-up at the age of 2 years revealed a weight of 10.9 kg in the 10th percentile and a height in the 5th percentile. The investigations were ordered and are shown in Table 1. She is active and playful. She achieved stability on long-term parenteral nutrition, and her immunodeficiency significantly improved, as evidenced by normal levels of immunoglobulins and a decreased frequency of infections. However, she started developing complications such as portal hypertension and hypersplenism. The liver is unlikely to recover with conservative management and will eventually require transplantation. Currently, she is put on PN with a volume of approximately 120 ml/kg and is receiving an average of approximately 70 kcal/kg/day, with monthly blood tests for monitoring.

**Table 1:** Summary of findings from patient investigations

Patient Laboratory Results			
Category	Test	Result	Reference Range
Liver Investigations	Bilirubin	2.1 mg/dl (H)	0-1.2 mg/dl
	Alkaline phosphatase	384 U/L (H)	≤ 281 U/L
	SGPT (ALT)	56 U/L (H)	0–33 U/L
	Albumin	3.0 g/L (L)	3.8–5.4 g/L
	Prothrombin time	13.1 s (H)	10–11.4 s
	INR	1.28 (H)	0.8–1.1
FBC Trend	WBC count	5.3 10^3/µL	5–15 10^3/µL
	RBC count	3.2 10^6/µL (L)	4-5.2 10^6/µL
	Hemoglobin	8.7 g/dL(L)	11.1–14.1 g/dL
	Hematocrit	26.4%(L)	30–38%
	RDW	23% (H)	11.5–14%
	Platelets count	57 10^3/µL (L)	200–490 10^3/µL
Urea and Electrolyte	Sodium	133 mmol/L	135–145 mmol/L
	Potassium	4.3 mmol/L	3.5-5.0 mmol/L
	Bicarbonate	18.9 mmol/L	22–28 mmol/L
	Urea	20 mg/dL	7–20 mg/dL
	Anion gap	5 mmol/L	3–11 mmol/L
Immunology	Anti-Nuclear Antibody	Positive 1/640(H)	Negative
	Anti-smooth Muscle AB	Negative	Negative
	Anti-liver kidney Microsomal	Negative	Negative
	ESR	Normal	0–20 mm/hr
	CPK	Normal	20–200 U/L
	Lymphocytes Absolute	2.0 10^3/µL	(6–9.0 10^3/µL)
Immunoglobulins (before Intravenous Immunoglobulin (IVIG))	Immunoglobulin G1 (IgG1)	0.4 g/L (L)	(1.940–8.420 g/L)
	Immunoglobulin G2 (IgG2)	0.1 g/L (L)	(0.225–3.000 g/L)
	Immunoglobulin G3 (IgG3)	0.09 g/L (L)	(0.186–0.853 g/L)
	Immunoglobulin G4 (IgG4)	0.02 g/L	(0.005–0.784 g/L)
	Total IgG	0.9 g/L (L)	(3.31–11.64 g/L)
Immunoglobulins (after Intravenous Immunoglobulin (IVIG))	Immunoglobulin G1 (IgG1)	5.84 g/L	(1.940–8.420 g/L)
	Immunoglobulin G2 (IgG2)	1.59 g/L	(0.225–3.000 g/L)
	Immunoglobulin G3 (IgG3)	0.86 g/L (H)	(0.186–0.853 g/L)
	Immunoglobulin G4 (IgG4)	0.06 g/L	(0.005–0.784 g/L)
	Total IgG	13.48 g/L (H)	(3.31–11.64 g/L)

Immune status screen Normal T and B cell repertoire (normal CD4/CD8 ratio)

## Discussion

Trichohepatoenteric syndrome (THES) is a genetic disorder caused by autosomal recessive mutations in the SKIC2 or SKIC3 genes that impact the SKI complex, which is essential for RNA surveillance. SKIC3 mutations are prevalent in THES patients, while SKIC2 mutations, accounting for approximately one-third of cases, often lead to more severe outcomes [4]. In our case, we conducted whole-exome sequencing (WES), which identified a homozygous variant (c.4070del, p.Pro1357Leufs*10) in SKIC3 (OMIM:614,589). This variant results in a frameshift, leading to a premature stop codon and subsequent mRNA degradation (nonsense-mediated decay) or protein truncation. The allele frequency of this variant in the general population has not been documented. Based on the available information, the variant is classified as likely pathogenic, causing autosomal recessive trichohepatoenteric syndrome 1 (THES1; OMIM: 222,470). Parental studies were performed, and the participants were found to be carriers of the same variant. Several other genetic tests, such as karyotype, aCGH and FISH for Digeorge were performed, and the results were negative.

It is characterized by three major presentations commonly observed in the majority of patients: diarrhea, dysmorphism, and hair changes. In a study involving 96 individuals with THES conducted by Bourgeois et al., all participants exhibited intractable diarrhea; 90% displayed woolly, brittle, and easily removable hair anomalies; and 84% presented facial dysmorphism, including a broad forehead, wide nasal root, and hypertelorism. [5] In our specific case, the patient presented with intractable diarrhea alongside facial dysmorphisms, featuring a broad forehead, mild facial asymmetry, thin and sparse hair and eyebrows, relatively large ears, thin lips, and a downturned angle of the mouth.

Diarrhea in THES commonly presents between 4 and 5 months of age [6]. However, our patient exhibited diarrhea from the first day of life, which is considered uncommon. Therefore, it is crucial to insist on the significance of including THES in the differential diagnosis for infants who present with persistent diarrhea from birth. Additional indicators of trichohepatoenteric syndrome (THES) include preterm delivery and intrauterine growth restriction (IUGR). THES patients typically exhibit a median gestational age of 35 weeks, as reported by Yang Lee et al., 2023 [7]. Comparing the two mutated genes, SKIC3 and SKIC2, a study by Bourgeois et al., 2018 revealed significantly greater birth weights and height percentiles for SKIC3 patients, with medians of 17.24 and 19.89, respectively [5]. However, our patient, born at 35 weeks, exhibited a weight percentile below 1, which does not match the study findings.

Hepatic involvement is also considered common, but it varies from transaminitis and hepatosplenomegaly to liver fibrosis [7]. It was noted that it is more frequently observed in patients with SKIC2 mutations (88%) than in those with SKIC3 mutations (51%) [5]. However, our patient presented with hepatosplenomegaly that progressed to liver failure. In addition, she developed portal hypertension and varices, which are considered uncommon in patients with SKIC3 mutations and were described in one patient with SKIC2 mutations [8].

Other less common presentations, including immunodeficiency, skin abnormalities, and congenital heart defects, were all present in our patient [5].

Kidney involvement is rarely mentioned in THES and is limited to small kidneys and polycystic kidneys [9]. Nevertheless, our patient presented with persistently low phosphate despite adequate supplementation. High urinary phosphate loss was noted to be due to tubular dysfunction, which has never been mentioned in previous studies. Normal serum phosphate levels were achieved after maximizing the phosphate concentration in the PN.

In this case, the patient experienced persistent hypoglycemia necessitating continuous monitoring and intervention. A similar manifestation involving a SKIC3 mutation, potentially linked to mitochondrial dysfunction induced by the genetic mutation, was previously documented in a single patient. Nevertheless, further investigations are needed for a comprehensive understanding [10].

Patients with THES exhibit survival rates of 93.3%, 86.7%, 74.3%, and 61.9% at 1 year, 5 years, 10 years, and 15 years, respectively [11]. While prior studies attributed mortality to infections (11;7), our case underscores liver deterioration as the primary prognostic factor. The primary management for THES remains PN and immunoglobulin supplementation, with antibiotics, steroids, immunosuppressants, and HSCT (hematopoietic stem cell transplantation) not recommended due to a lack of evidence for efficacy [3]. Our patient required monthly IVIG alongside long-term PN. Although multiorgan transplantation is the ultimate treatment, its unavailability currently stems from a high complication rate [8]. Rahmbia et al. (2017) reported complex liver, pancreas, and small bowel transplantation resulting in graft-versus-host disease (GVHD) and eventual mortality. Notably, younger patients and those with preexisting immunodeficiency face elevated risks of transplant failure [12].

## Conclusion

This case report describes a homozygous variant (c.4070del, p. Pro1357Leufs*10) in the SKIC3 gene. While she presented with classical features such as intractable diarrhea, facial dysmorphism, and sparse hair, her case exhibited several atypical findings, including early-onset diarrhea, severe liver involvement with portal hypertension and tubular dysfunction, and persistently low phosphate. These unique aspects could be due to this homozygous mutation. This case also underlines the importance of considering THES-type 1 in differential diagnoses even for atypical presentations. Further research is needed to better understand the relationship between genotypes and phenotypes and to explore other treatment options, particularly for severe liver complications.

## Data Availability

The data that support the findings of this study are available from the corresponding author upon reasonable request.

## Declarations

### Confirmation of the origin and exclusivity of publication

I hereby confirm that the manuscript is an original work that has not been published previously, nor is it currently under consideration for publication elsewhere.

## Abbreviations

THES: Trichohepatoenteric syndrome
SKIV2L: Superkillerviralicidic activity 2
TTC37: Tetratricopeptide repeat domain-containing protein 37
IUGR: Intrauterine growth restriction
SD/THE: Syndromic diarrhea/Tricho-Hepato-Enteric Syndrome
WES: Whole-exome sequencing
PN: Parenteral nutrition
HSCT: Hematopoietic stem cell transplantation
IVIG: Intravenous immunoglobulin
GVHD: Graft-versus-host disease

## References

[1] Bourgeois P, Esteve C, Chaix C, Béroud C, Lévy N, Fabre A, Badens C (2018). Tricho-Hepato-Enteric syndrome mutation update: mutations spectrum of TTC37 and SKIV2L, clinical analysis and future prospects. Hum Mutat.

[2] Hu X, Hu W, Sun X, Chen L. (2022). Novel TTC37 mutations in a patient with Trichohepatoenteric syndrome: a case report and literature review. In Translational Pediatrics (Vol. 11, Issue 6, pp. 1050–1057). AME Publishing Company. 10.21037/tp-21-574. 10.21037/tp-21-574 PMC9253954 35800280

[3] Alghamdi MS, Alsulimani EF, Almasrahi AM, Alsalmi KA. A case of mild trichohepatoenteric syndrome with new variant mutation in SKIV2L Gene: Case Report. Cureus.; 2021. 10.7759/cureus.19404. 10.7759/cureus.19404 PMC8654094 34926006

[4] Bourgeois P, Coste ME, Roman C, Barlogis V, Badens C. (2017). Management of syndromic diarrhea/tricho-hepato-enteric syndrome: A review of the literature. In Intractable and Rare Diseases Research (Vol. 6, Issue 3, pp. 152–157). International Advancement Center for Medicine and Health Research. 10.5582/irdr.2017.01040. 10.5582/irdr.2017.01040 PMC5608923 28944135

[5] Dorum S, Gorukmez O. (2021). Expanding the clinical spectrum in trichohepatoenteric syndrome. American Journal of Medical Genetics, Part A, 185(10), 2873–2877. 10.1002/ajmg.a.62354. 10.1002/ajmg.a.62354 34037310

[6] Kotecha UH, Movva S, Puri RD, Verma IC (2012). Trichohepatoenteric syndrome: founder mutation in Asian indians. Mol Syndromol.

[7] Lee KY, Bremner R, Hartley J, Protheroe S, Haller W, Johnson T, Whyte L (2023). Long term outcomes in children with trichohepatoenteric syndrome. Am J Med Genet Part A.

[8] Xinias I, Mavroudi A, Mouselimis D, Tsarouchas A, Vasilaki K, Roilides I, Lacaille F, Giouleme O (2018). Trichohepatoenteric syndrome: a rare mutation in SKIV2L gene in the first Balkan reported case. SAGE Open Med Case Rep.

[9] Fabre A, Bourgeois P, Chaix C, Bertaux K, Goulet O, Badens C, Trichohepatoenteric S, Adam MP, Feldman J, Mirzaa GM, Pagon RA, Wallace SE, Bean LJH, Gripp KW, Amemiya A (2018). Jan 11. GeneReviews® [Internet].

[10] Gao J, Hu X, Hu W, Sun X, Chen L. (2022). Novel TTC37 mutations in a patient with Trichohepatoenteric syndrome: a case report and literature review. In Translational Pediatrics (Vol. 11, Issue 6, pp. 1050–1057). AME Publishing Company.10.21037/tp-21-574. 10.21037/tp-21-574 PMC9253954 35800280

[11] Fabre A, Breton A, Coste ME, Colomb V, Dubern B, Lachaux A, Lemale J, Mancini J, Marinier E, Martinez-Vinson C, Peretti N, Perry A, Roquelaure B, Venaille A, Sarles J, Goulet O, Badens C (2014). Syndromic (phenotypic) diarrhoea of infancy/tricho-hepato-enteric syndrome. Arch Dis Child.

[12] Rambhia PH, Hanna R, Bergfeld WF (2018). Graft versus host disease in a pediatric multiple organ transplant recipient with trichohepatoenteric syndrome – a unique case report. Int J Dermatol.

